# Spatial transcriptomics in ovarian biology technologies: computational challenges, and biological insights

**DOI:** 10.1093/reprod/xaag079

**Published:** 2026-07-27

**Authors:** Ruixu Huang, Brittany A Goods

**Affiliations:** Thayer School of Engineering, Dartmouth College, Hanover, NH, United States; Faculty of Science, School of BioSciences, The University of Melbourne, Melbourne, Victoria, Australia

**Keywords:** ovary, single-cell RNA sequencing, spatial transcriptomics, computational biology

## Abstract

**In brief:** This review synthesizes the technical landscape of current spatial transcriptomic platforms, addresses computational challenges unique to ovarian tissue, and surveys biological discoveries across ovarian development, aging, follicle dynamics, and cancer, providing a practical framework to guide platform selection and analytical strategy in reproductive biology.

**Abstract:** The ovary is a structurally complex organ whose function depends on precisely coordinated interactions among multiple cell types. Spatially resolved transcriptomics (ST) has emerged as a powerful complement to single-cell RNA sequencing (scRNA-seq), enabling gene expression profiling within intact tissue and preserving the spatial context that dissociation-based methods inherently lack. This review provides a comprehensive overview of the major ST platforms, including sequencing-based technologies (Visium, Visium HD, Stereo-seq, and GeoMx) and imaging-based technologies (Xenium, MERSCOPE, and CosMx), with a focus on their distinct technical features, resolution trade-offs, and suitability for ovarian research. We survey 40 published studies applying ST to ovarian biology, spanning atlases, ovarian aging, follicle development and ovulation, and ovarian cancer. We also discuss typical computational analyses as well as their challenges specific to ovary, including cell segmentation of morphologically diverse cell populations, deconvolution of mixed-cell capture spots in sequencing-based platforms, quality control, batch correction, and spatially aware downstream analyses encompassing trajectory inference, cell–cell interaction modeling, gene regulatory network reconstruction, and more. Across these biological contexts, multimodal integration, pairing ST with scRNA-seq, spatial proteomics, or chromatin accessibility profiling, has proven increasingly valuable for resolving the full molecular complexity of ovarian biology. Nevertheless, some challenges persist, and no single platform is universally optimal for all research questions. Thoughtful alignment between biological objectives, tissue scale, and platform capability will be critical for advancing ST from descriptive mapping toward mechanistic and clinically translatable discovery.

Spatially resolved single-cell technologies have significantly advanced the study of reproductive biology by enabling direct mapping of gene expression onto intact ovarian tissue. In contrast to single-cell RNA-sequencing (scRNA-seq), which requires dissociation and therefore eliminates tissue context, spatial omics approaches retain the architectural information of the ovary, allowing molecular programs to be interrogated within their native microenvironment. This is crucial for properly contextualizing cells in ovarian tissues, enabling more advanced analyses than traditional scRNA-seq. This shift is particularly consequential for the ovary, where follicle development, ovulation, and luteinization occur through tightly coordinated interactions between oocytes, granulosa cells, theca cells, and surrounding stromal and immune populations. These structural features also change across menstrual or estrous cycles, with age, and in the context of disease. The ability to examine these changes in the context of the entire tissue environment provides an unprecedented lens for understanding ovarian biology. This review will summarize each spatial technology, highlight the ovarian features captured by each, and discuss how these technologies have already been used in the ovary. We will then discuss some computational challenges specific to this tissue, including accurate cell segmentation and delineation of follicle structures. Finally, we will discuss newer approaches that couple protein measurements along with single-cell transcriptomes as a powerful way to understand ovarian cells with greater depth.

## Technical overview of spatial omics platforms

**Figure 1:**
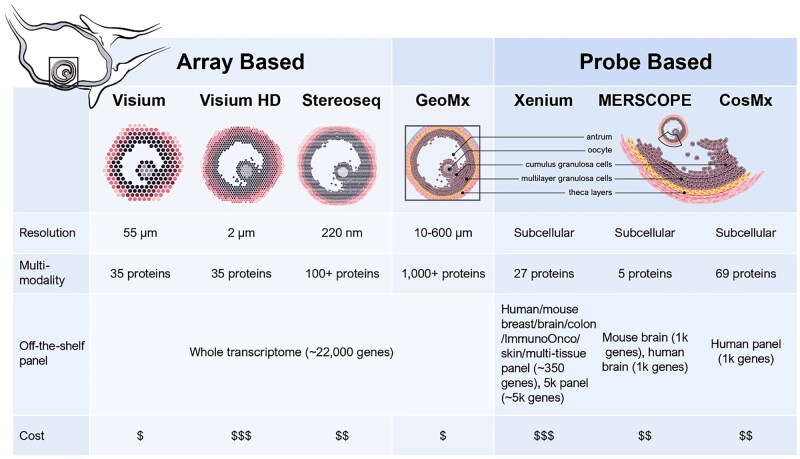
Summary of key technical features across commonly used spatial transcriptomics technologies. Sequencing-based spatial transcriptomics (sST) (array-based) captures transcripts using spatially indexed surfaces, typically enabling transcriptome-wide profiling at spot-level resolution. Imaging-based spatial transcriptomics (iST) (Xenium, MERSCOPE, CosMx) relies on in-situ hybridization and optical barcoding to detect subcellular transcripts within intact tissues. Resolution indicates approximate spatial capture scale or effective cellular resolution. GeoMx is presented separately as a region of interest (ROI)-based platform that does not fit neatly into either category, offering user-defined spatial selection with flexible resolution. Multimodality reflects the availability of integrated protein detection. Off-the-shelf panels denote the availability of predefined gene panels versus whole-transcriptome capability. Cost categories are relative and intended for conceptual comparison rather than exact pricing. This comparison highlights trade-offs among spatial resolution, transcriptome breadth, multiplexing capacity, and platform scalability. Multimodality values indicate the maximum number of proteins detectable alongside RNA in the same tissue section using each platform’s integrated multiomics workflow, based on manufacturer specifications at time of publication. For sST platforms, Visium and Visium HD support simultaneous detection of 35 proteins via the CytAssist Gene and Protein Expression assay (10x Genomics. Visium CytAssist Spatial Gene and Protein Expression); Stereo-seq supports detection of 100+ proteins via Stereo-CITE, which integrates CITE-seq with the Stereo-seq workflow (STOmics) (STOmics. Stereo-CITE Proteo-Transcriptomics Solution, 2026); and GeoMx supports ROI-based profiling of 570+ proteins (Bruker Spatial Biology. GeoMx Protein Assays, 2026). For iST platforms, Xenium supports codetection of 27 proteins via its integrated protein subpanels (10x Genomics. Xenium In Situ Gene and Protein Expression, 2026); MERSCOPE supports up to six proteins via its Protein Co-Detection Kit (Vizgen. Protein Co-Detection with MERSCOPE, 2026); and CosMx supports detection of 72 proteins via its Same-Cell Multiomics workflow (Bruker Spatial Biology. CosMx Same-Cell Multiomics, 2026). (Figure provided by SciStories.)

Spatial omics technologies have evolved significantly in the past few years (Cheng et al., 2023; Moses & Pachter, 2022) and several commercial platforms with distinct features that shape their utility in the context of ovarian research (Figure 1) are now available. The general chemistries and technology innovations that have enabled these technologies have been discussed in depth elsewhere (Marx, 2021; Moses & Pachter, 2022; Robles-Remacho et al., 2023); thus, we will focus on summarizing their utility in the context of the ovary. In general, spatial transcriptomic (ST) technologies can be categorized into two major approaches: sequencing-based (sST) and imaging-based (iST) (Dezem et al., 2024). Sequencing-based platforms, including Visium (Ståhl et al., 2016), Visium HD (Oliveira et al., 2025), GeoMX (Merritt et al., 2020), and Stereo-seq (Chen et al., 2022), capture RNA molecules in situ on barcoded arrays, enabling whole-transcriptome profiling at lower spatial resolution but with full-depth transcript coverage. In contrast, imaging-based methods, such as MERSCOPE (Chen et al., 2015), CosMx (He et al., 2022), and Xenium (Janesick et al., 2023) rely on repeated hybridization and imaging of fluorescently labeled probes to detect predefined gene panels with subcellular spatial resolution. While iST enable single-cell resolution, sST can profile many genes more cost-effectively (Virtanen et al., 2025). Notably, Stereo-seq achieves a feature resolution of 220 nm, approaching the subcellular precision of iST platforms, but remains array-based, meaning that transcript assignment still relies on spatial binning rather than cell boundary-defined segmentation, and mixed-cell signals can persist even at this resolution. We will provide a brief overview of each approach, focusing on the advantages and types of ovarian features captured by each method.

### Sequencing-based spatial transcriptomic approaches

The sST technologies were the first to gain widespread adoption, emerging naturally from the conceptual and technical foundations of scRNA-seq (Ståhl et al., 2016). Building upon the well-established principles of RNA capture, barcoding, and next-generation sequencing, these methods extended transcriptional profiling into the spatial dimension by immobilizing barcoded oligonucleotides directly onto solid surfaces. Although early imaging-based approaches, such as seqFISH (Eng et al., 2019; Lubeck et al., 2014), appeared around the same time, sequencing-based technologies were the first to be commercialized, likely because they aligned closely with existing sequencing workflows and data analysis frameworks familiar to the single-cell community.

Among these, 10x Genomics Visium remains the most widely used platform. Each Visium slide contains four imaging areas, each measuring 6.5 × 6.5 mm, densely arrayed with 55 µm-diameter capture spots spaced 100 µm apart. Each spot contains spatially barcoded oligo (dT) probes that hybridize to mRNA transcripts, which are then reverse-transcribed and sequenced (10x Genomics Visium Spatial Assays product page). The resulting data preserve both transcript identity and spatial coordinates, enabling whole-transcriptome, unbiased profiling across large tissue sections. Visium is compatible with both fresh-frozen and formalin-fixed paraffin-embedded (FFPE) specimens, making it particularly versatile. In practice, the 6.5-mm capture area is sufficient to accommodate an entire mouse ovary, although typically not a human one, and allows visualization of large-scale tissue organization throughout the whole organ. However, the relatively coarse resolution means that each spot may capture transcripts from multiple adjacent cells, such as granulosa and theca cells within a follicle, potentially confounding fine-grained interpretation. Coupled with computational challenges, this can significantly hinder cell-type identification and downstream analyses of these datasets, making it challenging to distinguish closely related cell lineages, such as cumulus and granulosa cells, or theca and stromal cells (Martinez et al., 2025; Zhao et al., 2025a).

Visium HD is the newest version of 10x Genomics’ sST platform. While it retains the core chemistry of the original Visium platform, it introduces a dramatically finer capture grid (10X Visium HD Product). Each Visium HD slide maintains the same imaging area of 6.5 × 6.5 mm, but the tissue is profiled through a dense array of 2-µm capture spots. As a result, this approach achieves near–single cell spatial resolution while enabling whole-transcriptome coverage. Visium HD is also compatible with FFPE and fresh-frozen tissues, thereby significantly enhancing spatial granularity in complex reproductive tissues. Deconvolution for cell-type identification is still needed for Visium HD, since the bins do not exactly match cell boundaries (Oliveira et al., 2025). This means some of the core challenges outlined above for Visium still remain, along with challenges in computational management, given the sheer volume of data generated by a single Visium HD sequencing run, which can reach up to hundreds of gigabytes.

Another sequencing-based platform, Stereo-seq (Beijing Genomics Institute Genomics), employs a distinct molecular architecture to achieve both high spatial resolution and large imaging areas (Stereo-Seq Product Page). Instead of circular capture spots, Stereo-seq utilizes a patterned array of DNA nanoballs that carry spatial barcodes incorporating coordinate identifiers, molecular identifiers, and poly(dT) sequences. This design enables a feature resolution of 220 nm with minimal spacing, offering subcell resolution across an imaging field up to 130 × 130 mm, representing the largest capture area to date. This is useful because it can fit multiple sections of the human ovary on a single slide. Stereo-seq is also compatible with fresh-frozen and FFPE tissues and has demonstrated robust performance in whole-organ mapping, including embryonic and ovarian structures (Chen et al., 2022).

The GeoMx Digital Spatial Profiler (Bruker Spatial Technology) bridges sequencing- and probe-based approaches (Merritt et al., 2020). Rather than capturing the entire tissue, GeoMx relies on user-defined regions of interest (ROIs) that span a total imaging area of 35.3 × 14.1 mm and uses photocleavable oligo-conjugated probes to profile RNA targets. The released oligos are collected and quantified by next-generation sequencing, yielding digital counts analogous to those obtained from sequencing-based data. While GeoMx lacks subcellular resolution and does not provide truly whole-transcriptome coverage (depending on whether a targeted or whole-transcriptome panel is used), it remains a valuable tool for studies focused on histologically defined ovarian compartments where targeted spatial quantification is prioritized over single-cell resolution.

From a practical standpoint, sequencing-based spatial methods share a common challenge: their spatial resolution. In the ovary, where neighboring cell layers, such as granulosa and theca cells, are tightly packed together, each capture feature may contain a mixture of cell types, making it challenging to assign signals with single-cell precision. The ovary also contains cell types that are particularly challenging to resolve using current sST. Ovarian surface epithelial cells, which form a single layer surrounding the mouse ovary and give rise to multiple subtypes of ovarian cancer, are difficult to capture with single-cell precision given their sparse spatial distribution. Diffuse immune cells and specialized cumulus cells present similar challenges due to their rarity and scattered localization across the tissue. Oocytes, by contrast, present a different type of challenge. Mammalian oocytes vary substantially in size across species, ranging from approximately 80 µm in mice to over 120 µm in humans, and their large diameter means they often span multiple capture spots, resulting in diluted and incomplete transcriptional signals rather than a clean single-cell profile. Beyond resolution, their low abundance within any given tissue section further limits robust profiling, as only a single oocyte is present per follicle. Nevertheless, these technologies excel in transcriptome-wide discovery and serve as good platforms for constructing tissue atlases and generating hypotheses, particularly when the goal is to capture the global transcriptional organization across large tissue contexts.

### Imaging-based spatial transcriptomic approaches

Probe-based, or iST, technologies were developed contemporaneously with sequencing-based methods, offering a complementary route to higher-resolution mapping and direct visualization of transcripts within individual cells (Wang et al., 2023). These methods use fluorescently labeled oligonucleotide probes that bind to specific target transcripts in situ, followed by microscopy-based imaging to record the positions of these transcripts. Conceptually, this approach trades whole-transcriptome coverage for single-cell resolution by using a targeted transcript panel. These technologies leverage advanced super-resolution and multiplexed fluorescence microscopy; thus, each iST approach depends on specialized imaging instruments and optical workflows unique to each platform.

Commercially available iST platforms include Xenium (10x Genomics) (Janesick et al., 2023), MERSCOPE (Vizgen) (Chen et al., 2015), and CosMx (Bruker Spatial Technology) (He et al., 2022). Each system provides subcellular spatial localization of transcripts across imaging areas sufficiently large for whole-mouse-ovary profiling, approximately 10.45 × 22.45 mm for Xenium, ∼16.88 × 20.98 mm for MERSCOPE, and ∼20 × 15 mm for CosMx. These field sizes are especially advantageous in reproductive biology, where compartmentalized architecture can be captured for the entire mouse ovary. Beyond their imaging footprints, these platforms also differ in their computational pipelines, such as spot calling, transcript assignment, and cell segmentation (Chen et al., 2023). These differences can significantly impact downstream analyses and data integration with scRNA-seq or sequencing-based spatial datasets (see “Current computational analysis approaches, challenges, and limitations”).

A shared limitation of all iST platforms is their reliance on targeted gene panels. Effective study design requires careful inclusion of ovarian lineage markers, pathway components, and ligand–receptor pairs, balanced against the risk of saturating signals from highly abundant transcripts. Although ovarian scRNA-seq resources can guide panel construction (Huang et al., 2025), gene panel representation remains uneven across many cell states and developmental processes relevant to folliculogenesis, ovulation, and luteinization. However, platforms are rapidly expanding the number of genes offered in their panels and custom panels. For example, as of writing this paper, MERSCOPE Ultra now accommodates panels with up to 1,000 genes, and 10x Genomics has announced the Xenium 5k Prime panel, which can also accommodate an additional 100 custom probes (MERSCOPE Ultra; Xenium 5k panel).

Unfortunately, most of these probe sets are not designed to meet the needs of ovarian science. Xenium has several predesigned human panels, including those for human breast (280 genes), human brain (266 genes), human colon (322 genes), human immune-oncology (380 genes), human lung (289 genes), human multi-tissue (377 genes), human skin (260 genes), mouse brain (247 genes), and mouse multi-tissue panel (379 genes). MERSCOPE offers a mouse brain panel (1,000 genes) and a human brain panel (1,000 genes). CosMx has one human panel (1,000 genes). We compared these off-the-shelf options against a curated list of 200 gene markers covering all major murine ovarian cell types (Morris et al., 2022). The CosMx human panel showed the highest overlap with 50 genes, followed by MERSCOPE’s mouse brain panel (37 genes) and Xenium’s 5k panel (78 genes). This underscores the limitations of predesigned panels for ovarian studies, suggesting that panel customization is essential to ensure sufficient marker coverage for accurate downstream cell typing. If reproductive cell–type markers are underrepresented in the panel, specific populations may be unidentifiable and effectively missing from downstream analyses. Conversely, building a panel with markers for a dominant cell type can be equally detrimental. In regions with densely packed cells, such as luteal cells in the corpus luteum, an excess of high-expression probes can lead to optical crowding and signal saturation. This phenomenon can overwhelm the iST resolution and compromise data integrity.

Although often necessary, customizing the panel can be difficult because targeted panel design is constrained by reliance on prior biological knowledge. Unlike whole-transcriptome sequencing, targeted iST methods require researchers to select gene panels beforehand, necessitating a deep understanding of the biology to be measured. In our prior work using MERSCOPE, we successfully designed a custom ovary panel using existing transcriptomic data (Huang et al., 2025). Thus, designing balanced panels requires a multipronged approach, including cross-referencing existing single-cell transcriptomic and bulk RNA-seq repositories to ensure the probe set can resolve tissue heterogeneity without inducing signal failure.

Together, iST technologies deliver true single-cell and subcellular resolution, enabling detailed visualization of follicular structures, spatial relationships among granulosa, theca, stromal, and immune populations, and localization of key transcripts within tightly organized ovarian compartments (Wagner et al., 2020). However, their targeted nature limits their suitability for discovery-driven or atlas-scale efforts unless additional probes are included in experiments. Although ovarian scRNA-seq atlases are increasingly comprehensive, many processes, such as rare cell states, transitional phenotypes, immune cells in the ovary, and poorly characterized pathways, remain insufficiently annotated for robust panel design. As a result, pilot studies, iterative panel refinement, and parallel scRNA-seq generation remain essential for researchers aiming to use iST platforms to explore novel or undercharacterized aspects of ovarian biology.

### Multimodal profiling

**Figure 2:**
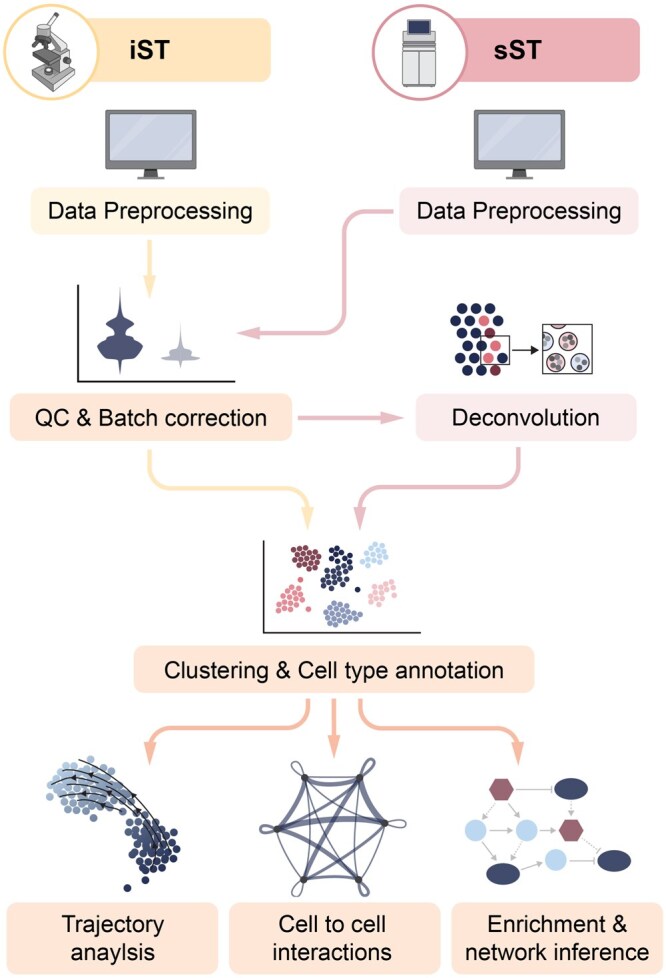
Schematic overview of computational workflows used to analyze imaging-based (iST) and sequencing-based (sST) spatial transcriptomic datasets. Both modalities undergo initial data preprocessing, including data preprocessing, quality control (QC), and batch correction as needed. The sST datasets typically require deconvolution to resolve mixed-cell transcriptional signals within spatial capture regions. Outputs from both pipelines converge for downstream clustering and cell-type annotation, enabling more biological inference, including trajectory reconstruction, cell–cell interactions, and pathway or network enrichment analyses. Workflow colors indicate platform-specific pathways: yellow arrows denote the iST pipeline, and pink arrows denote the sST pipeline. In the iST workflow, data preprocessing is followed directly by QC and batch correction before downstream analysis. In the sST workflow, data preprocessing feeds into deconvolution to resolve mixed-cell spots, which then converges with the iST pipeline at the QC and batch correction step prior to clustering and cell-type annotation. Downstream analyses are applicable to both platforms and should be interpreted with spatial context in mind; in the context of ovarian biology, these include follicle-resolved trajectory inference, spatially grounded cell–cell interaction analysis within defined follicular compartments, and pathway enrichment within morphologically annotated tissue regions. (Figure Provided by SciStories).

Recent ovarian ST studies have already demonstrated that transcript-only approaches have limitations, prompting many researchers to layer additional data types to strengthen interpretation (Schweizer et al., 2025). In our survey of 39 studies, almost half pair ST with another modality, with over a third choosing scRNA-seq or single-nucleus RNA sequencing, and less frequently a second spatial platform, spatial proteomics, or ATAC-seq (Figure 2). Those without their own multimodal datasets often rely on external single-cell atlases for additional interpretation. The ability to quantify RNA and protein within the same tissue section provides critical complementary information, particularly in ovarian biology, where transcript–protein correspondence is imperfect and protein-level measurements can reveal functional states and signaling dynamics that cannot be inferred from RNA alone (Buccitelli & Selbach, 2020; Liu et al., 2016). Several platforms now support true same-section multiomics, including CosMx, Xenium, Visium CytAssist, and Stereo-seq, although they vary in chemistry and proteomic plexity. Recent comparative reviews, including this one, offer guidance for selecting technologies aligned with specific research aims (Lee et al., 2025; L. Liu et al., 2024). Together, these trends underscore the central role that multimodality is beginning to play in the generation, interpretation, and application of spatial data across ovarian research.

**Table 1:** Platform recommendation framework for ST in ovarian biology.

Biological question	Recommended platforms	Key considerations
**Whole-transcriptome discovery/tissue atlas construction**	Any sST	Whole-transcriptome coverage without prior gene selection; suitable for unbiased hypothesis generation; compatible with FFPE and fresh-frozen tissues
**Single-cell resolution cell-type characterization**	Any iST	Custom panel design required; off-the-shelf panels have limited ovarian marker coverage; segmentation quality critical; pair with scRNA-seq reference for annotation
**Rare cell-type identification (immune cells, OSE, etc.)**	Any iST	iST essential for spatial localization of sparse populations; panel must include sufficient markers for rare cell types; risk of panel saturation from highly abundant cell types
**Trajectory inference, developmental or time-course studies**	Any sST or iST	Histological context enables morphology-guided follicle stage assignment; multiple time points required for dynamic processes such as ovulation; pseudotime tools appropriate for developmental trajectories; RNA velocity preferred for capturing rapid transcriptional dynamics; within-follicle gradients may be more informative than global tissue axes
**Large tissue area profiling**	Stereo-seq, GeoMx	Stereo-seq offers the largest capture area and approaches subcellular resolution; GeoMx supports ROI-based profiling across large tissue sections
**Tumor microenvironment/­ovarian cancer**	Any iST	FFPE compatibility critical for archival clinical samples; immune and stromal cell markers must be represented in panel since those are usually considered relevant
**Targeted hypothesis testing in specific compartments**	Any iST	Single-cell and subcellular resolution critical when panel design is already established; iST enables precise spatial localization within defined compartments

This table provides a practical guide for selecting ST platforms based on the primary biological question of interest. Recommended platforms are categorized as sST or iST. ST, spatial transcriptomics; sST, sequencing-based spatial transcriptomics; iST, imaging-based spatial transcriptomics; ROI, region of interest; FFPE, formalin-fixed paraffin-embedded; scRNA-seq, single-cell RNA sequencing; OSE, ovarian surface epithelial.

Among imaging-based technologies, CosMx recently introduced a Same-Cell Multiomics workflow which enables RNA profiling and up to 72 proteins from the same FFPE section at subcellular resolution (Bruker Spatial Biology. CosMx Same-Cell Multiomics, 2026). Xenium similarly supports RNA–protein codetection using fluorescently barcoded antibodies, currently validated for approximately 27 proteins, with options for custom additions (10x Genomics. Xenium In Situ Gene and Protein Expression, 2026). In contrast, MERSCOPE remains primarily RNA-focused, with protein detection limited to low-plex immunofluorescence costaining rather than a fully integrated, high-plex workflow, and provides up to six proteins with its protein staining reagent kit (Vizgen. Protein Co-Detection with MERSCOPE, 2026). Sequencing-based technologies are also expanding multimodal capability. Visium CytAssist and Visium HD CytAssist enable simultaneous whole-transcriptome RNA profiling and multiplexed protein detection using oligo-tagged antibodies, with up to 35 prevalidated antibody panels available, as well as customization (10x Genomics. Visium CytAssist Spatial Gene and Protein Expression). Stereo-seq extends to protein detection via Stereo-CITE-seq, enabling simultaneous RNA–protein profiling with antibody-derived tags (STOmics. Stereo-CITE Proteo-Transcriptomics Solution, 2026). Across these platforms, plexity varies substantially, reflecting active development across the field.

GeoMx Digital Spatial Profiler occupies a distinct position as a flexible ROI-based multimodal system capable of profiling the entire transcriptome and more than 570 proteins, either separately or in combined workflows (Bruker Spatial Biology. GeoMx Protein Assays, 2026). Although its resolution is limited compared with single-cell imaging platforms, the ability to measure high-plex protein panels across histologically defined ovarian regions makes it well-suited for targeted clinical or translational studies, particularly in human ovarian tissue, where specific compartments or pathological features may be of interest.

Beyond RNA–protein codetection, several emerging technologies are beginning to extend spatial biology into additional molecular layers. Deep visual proteomics, for instance, integrates high-resolution imaging with mass spectrometry-based proteomic profiling to quantify hundreds of proteins (Mund et al., 2022). Although still technically demanding and not yet widely applied in ovarian research, this approach offers a means to validate hypotheses that cannot be captured by transcriptomics alone. Similarly, spatial epigenomic modalities, including spatial ATAC-seq and spatial CUT&Tag, are now being used to map chromatin accessibility and regulatory element activity within intact tissues (Deng et al., 2022; Llorens-Bobadilla et al., 2023). Early studies applying these tools demonstrate their potential to reveal how lineage commitment, steroidogenic transitions, or pathological remodeling are encoded at the chromatin level (Lee et al., 2025; X. Liu et al., 2024; Zhang et al., 2023). However, such applications remain limited and warrant further exploration in ovarian tissues.

## Current computational analysis approaches, challenges, and limitations

A methodological dichotomy between sST and iST currently defines the computational landscape for spatial omics analyses, with implications for the study of ovarian biology (Figure 2). While sST analysis has largely converged around well-established single-cell workflows adapted for spatial contexts, iST relies on diverse, image-centric Python ecosystems. The ovary is characterized by extreme morphological heterogeneity and unique cellular geometries (e.g., a range of cell sizes), which can make iST workflows challenging. The following sections outline the critical data-processing steps and the downstream analytical strategies used to decode ovarian physiology and pathology, highlighting their challenges and limitations.

### Before analysis for iST: data preprocessing

For high-resolution iST, the fundamental prerequisite for downstream analysis is converting raw image data into a quantitative gene-by-cell matrix. To achieve this, each platform maps transcripts to exact spatial coordinates (*x*, *y*) and assigns them to specific cells based on segmentation boundaries established via nuclear (4′,6-diamidino-2-phenylindole) and membrane staining (Moffitt et al., 2022). Given the massive scale of these datasets, often encompassing millions of cells, vendors typically provide proprietary, pretrained deep learning or machine learning models optimized for their specific hardware to perform automated cell segmentation (Jones et al., 2025). However, researchers can reprocess raw image data using open-source algorithmic frameworks rather than relying solely on vendor default pipelines. Popular tools for custom segmentation include Cellpose (Stringer et al., 2021), DeepCell (Mesmer) (Greenwald et al., 2022), and Baysor (Petukhov et al., 2022). The primary advantage of manual resegmentation is the flexibility it offers in adjusting parameters, which is critical when dealing with issues like variable staining quality. Suppose a specific morphological stain is faint or discontinuous. In that case, a common issue in lipid-rich ovarian tissue is that standard algorithms might fail to define accurate boundaries, leading to transcript miscounts between neighboring cells. By employing custom frameworks, researchers can adjust channel weights, such as reducing the reliance on a poor membrane stain in favor of a robust nuclear signal, to optimize the segmentation mask. This precise tuning ensures that transcripts are assigned to the correct cellular units, directly improving the fidelity of the final expression matrix.

Despite these methodological options, the ovary remains a challenging environment for both proprietary and open-source cell segmentation models. As noted in the above section, the large diameter of mammalian oocytes means their transcriptional signal is distributed across multiple spatial units (Jones et al., 2024). This poses an additional challenge for iST segmentation, where generic machine learning segmentation models have been shown to underperform on oocytes relative to oocyte-specific approaches. Methods that incorporate manual or digital selection of oocytes based on their distinctive morphology prior to segmentation and downstream analysis may be preferred, highlighting the need for morphology-aware segmentation in ovarian tissues (Fjeldstad et al., 2024).

### Before analysis for sST: data preprocessing

For sST, the preprocessing workflow remains conceptually similar to scRNA-seq, as the technology still relies on cDNA synthesis, library construction, and sequence alignment (10X Genomics Space Ranger 2.1; Ståhl et al., 2016). Raw outputs consist of sequencing reads tagged with spatial barcodes rather than explicit optical *x*–*y* coordinates. Spatial information is computationally reconstructed by assigning decoded barcodes to their predefined array positions, yielding gene expression matrices indexed by spatial capture units. Each platform provides its own preprocessing software: 10x Visium uses SpaceRanger (10X Genomics Space Ranger 2.1) for alignment and feature counting; GeoMx relies on the GeoMx NGS Pipeline (Merritt et al., 2020), and Stereo-seq employs pipelines such as SAW (Chen et al., 2022) for alignment and spatial binning. Despite differences in implementation, these pipelines all produce the same core output, a gene-by-location count matrix paired with spot-level coordinates and metadata.

The most significant limitation of sST preprocessing arises from the technology’s physical design: capture spots are mostly larger than individual cells. Whereas single-cell sequencing assigns reads to individual barcoded cells, sST platforms assign reads to capture areas that often encompass multiple cells. This mismatch between biological and technical resolution leads to another problem. Instead of representing a single cell type, each spot reflects a weighted mixture of the local cellular neighborhood, a pattern reported in various ovarian research (Le et al., 2026; Lu et al., 2024; Martinez et al., 2025; Russ et al., 2022; Zhao et al., 2025a).

This issue is particularly acute in ovarian tissue, especially fetal ovaries, where the spatial organization is both dense and functionally intricate. The cellular architecture of ovaries and fetal ovaries differ between species. In human fetal ovaries, follicles contain tightly packed pregranulosa and nascent theca layers, only a few micrometers apart; in mouse fetal ovaries, theca cells have not differentiated, and the tissue consists primarily of germ cells surrounded by pregranulosa cells embedded within stromal precursor populations. In both species, immune populations tend to be sparse, transient, or distributed across stromal regions. As a result, oversized capture areas can readily blur boundaries between follicular compartments, collapse signals from adjacent layers, and obscure the fine-scale heterogeneity essential for interpreting ovarian physiology. Mixed spots can also make it challenging to distinguish theca from granulosa cells or confound attempts to identify rare and diffuse populations, such as immune cells. Overall, each approach has challenges, and there are tradeoffs to balance when choosing a platform for spatial profiling in the ovary (Figure 1).

### Upstream analysis: quality check and batch correction

Quality control (QC) and batch correction are critical steps in ST, ensuring that downstream analyses reflect genuine biological variation rather than technical noise. Because sST and iST rely on fundamentally different chemistries, the QC parameters and the nature of batch effects differ between platforms. For sST technologies, QC metrics closely resemble those used in scRNA-seq. These typically include the number of detected genes per spot, total Unique Molecular Identifier (UMI) counts, the percentage of mitochondrial transcripts, and the identification of low-quality spots with insufficient complexity. In contrast, iST platforms use direct hybridization, iterative imaging, and fluorescence detection. Their QC metrics therefore emphasize image-derived parameters, including transcript counts per cell, the number of genes detected per cell, and geometric features such as cell area or signal density. Poor segmentation, excessive background fluorescence, or imaging artifacts are common reasons for cell removal (Petukhov et al., 2022). Quality control for iST primarily reflects optical signal quality and segmentation accuracy rather than transcriptional depth. In ovarian datasets, where granulosa layers are densely packed, and theca cells have elongated morphologies, segmentation-driven QC is critical to avoid cell merging or misassignment. Despite the importance of these steps, the majority of studies surveyed for this review provide only minimal or inconsistent descriptions of their QC procedures, highlighting a critical gap in reporting standards. As ovarian spatial omics expands, more explicit and rigorous documentation of QC criteria will be essential for ensuring reproducibility, comparability across datasets, and accurate biological interpretation.

Differences in chemistry also shape the nature of batch effects. The sST platforms typically exhibit more substantial experimental batch effects than iST platforms. For sST, variability in tissue permeabilization, cDNA synthesis, PCR amplification, and sequencing depth can introduce substantial technical heterogeneity across experiments. By contrast, iST platforms tend to exhibit lower experimental batch variability because they rely on direct probe hybridization and imaging rather than enzymatic amplification. Their iterative, barcoded detection cycles also contribute to more uniform signal profiles across libraries. However, both sST and iST share a common challenge: interindividual biological variability, a problem not unique to ST but inherent to any multisample omics studies. In human studies, variabilities driven by age, menstrual cycle stage, hormonal status, and inherent donor heterogeneity frequently dominate ovarian datasets. In mouse studies, analogous interanimal variability, including differences in estrous cycle stage, age, and genetic background, can similarly introduce heterogeneity across sample. Fortunately, these interindividual differences typically do not require specialized spatial correction tools; established single-cell methods remain effective. In current ovarian spatial studies, batch correction is most often performed using Harmony (Korsunsky et al., 2019), available in both Python and R, which integrates samples by aligning embeddings while preserving biological structure. Alternatives such as ComBat and single-cell Variational Inference (scVI) are also used (Johnson et al., 2007; Lopez et al., 2018). From an experimental design perspective, minimizing batch effects in spatial ovarian studies benefits from careful separation of biological and technical variables. Whenever possible, biological replicates should be distributed across slides, runs, and reagent lots to avoid confounding donor-specific effects with technical artifacts (Liu & Yang, 2024; Zhang and Hou, 2025; Ludington et al., 2026). Including a common control sample on each slide or run, when feasible, can further aid in identifying and normalizing technical variation. Consistent annotation of key covariates, such as age, menstrual cycle phase, and tissue processing parameters, facilitates downstream modeling and integration, enabling batch-aware analyses that preserve biologically meaningful spatial structure while minimizing technical noise (Liu & Yang, 2024; Zhang and Hou, 2025; Ludington et al., 2026).

### Upstream analysis: deconvolution and integration

**Figure 3:**
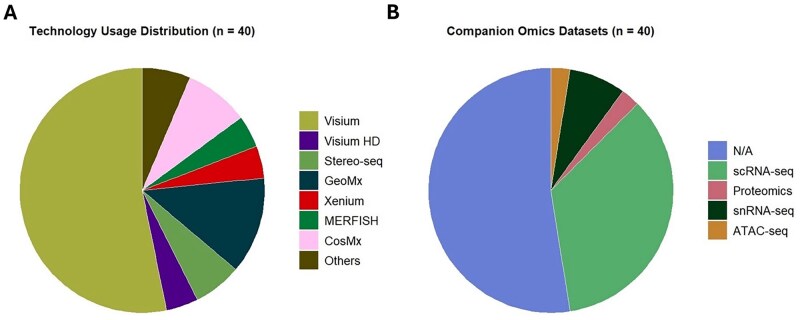
Distribution of spatial transcriptomic (ST) technologies used for each study surveyed for this review. (A) The graph shows that Visium is the most prevalent choice. (B) Distribution of the type of companion omics datasets generated for each study. N/A stands for none other than ST.

For sST, deconvolution is a computational strategy that infers the cellular composition of capture spots. Because sST platforms capture transcripts from areas larger than the diameter of a single cell, many spots contain RNA from multiple cell types. Deconvolution is therefore essential for separating overlapping signals and reconstructing fine-grained cellular organization, or for identifying rare populations masked by mixed spots. Several well-established tools are available, such as Cell2location (Kleshchevnikov et al., 2022), CARD (Ma & Zhou, 2022), Tangram (Biancalani et al., 2021), Robust Cell Type Decomposition (RCTD) (Cable et al., 2022), SPOTlight (Elosua-Bayes et al., 2021), and DestVI (Lopez et al., 2022), which benchmarking paper finds CARD, Cell2location, and Tangram to outperform others (H. Li et al., 2023). These methods differ in underlying modeling strategies, but most share a common requirement: they rely on high-quality scRNA-seq references. Without appropriate references, especially for populations that may share many common transcriptional programs, such as granulosa cells and cumulus cells, deconvolution will either have a compromised performance or systematically misrepresent these cells. The reliance on single-cell references means that including complementary scRNA-seq data for sST studies is crucial. Across the 40 studies surveyed for this review, more than a third generated complementary scRNA-seq datasets, either from matched tissues or from the same experimental system (Figure 3). The value of combining single-cell and spatial modalities extends beyond simple cross-validation. The growing trend reflects the field’s recognition that single-cell and spatial modalities provide mutually reinforcing layers of information: scRNA-seq offers molecular resolution, while ST supplies architectural context (Longo et al., 2021). Even subcellular-resolution spatial platforms remain constrained by transcript detection efficiency and spatially correlated noise. In contrast, dissociated scRNA-seq physically isolates individual cells prior to library preparation, eliminating segmentation ambiguity and spot-level mixing. Integrative strategies that deliberately leverage these complementary properties transform parallel measurements into a cohesive analytical framework, enabling better interpretation than either modality alone.

### Downstream analysis: clustering, cell type annotation, and differential expression

Clustering and cell type annotation are foundational analytical steps in ST, providing the unit on which all subsequent biological interpretation depends. Most ovarian studies rely on clustering frameworks developed initially for scRNA-seq. Seurat (R) (Stuart et al., 2019) and Scanpy (Python) (Wolf et al., 2018) dominate this landscape, offering integrated pipelines that include data normalization, dimensionality reduction (principal component analysis [PCA], uniform manifold approximation and projection [UMAP], t-distributed stochastic neighbor embedding [t-SNE]), nearest-neighbor graph construction, and graph-based clustering via Louvain or Leiden algorithms (Blondel et al., 2008; Traag et al., 2019). These platforms are popular because they are well-documented, computationally stable, and actively maintained. Both also provide extensive visualization capabilities, allowing users to map clusters onto UMAP embeddings, tissue coordinates, or histological images, and to generate spatial feature plots that reveal marker expression patterns across the ovary. Several platform-specific pipelines also include built-in clustering modules. GeoMx provides proprietary analysis workflows via the GeoMx NGS Pipeline that support clustering of ROIs. Stereo-seq similarly provides the SAW pipeline (Stereo-Seq Product Page), which includes spatial binning, normalization, and clustering. While these platform-native tools are convenient entry points, particularly for users without deep computational backgrounds, most researchers ultimately export data to generic toolkits to enable more flexible and sophisticated downstream analyses.

In parallel, a growing suite of methods has been developed specifically for ST clustering. Tools such as SpaGCN (Hu et al., 2021) and BayesSpace (Zhao et al., 2021) explicitly incorporate spatial coordinates, spatial adjacency, or histological features to refine domain detection; packages like Randomized Spatial PCA (RASP) provide a scalable, spatially aware dimensionality-reduction framework that enables rapid exploration of spatial structure prior to clustering (Gingerich et al., 2025). These approaches favor domains that are not only transcriptionally coherent but also anatomically contiguous, which may be particularly advantageous in the ovary, where follicular layers and stromal compartments exhibit a spatially organized pattern that may not be fully captured by expression similarity alone. Differential expression analysis (DEA) represents another foundational step in ST workflows, enabling the identification of genes that distinguish clusters and cell states. Common DEA implementations are embedded in Seurat (Butler et al., 2018) and Scanpy (Wolf et al., 2018), which rely on statistical tests such as the Wilcoxon rank-sum test (Wilcoxon, 1945), MAST (Finak et al., 2015), or negative binomial linear models (Anders & Huber, 2010). These approaches are effective for detecting transcriptional differences between predefined groups but do not explicitly model spatial structure. A growing class of methods has been developed to detect spatially variable genes directly from tissue coordinates. Tools such as SpatialDE (Svensson et al., 2018) use Gaussian process regression in gene expression to identify genes exhibiting structured spatial patterns across the tissue. Despite their power, these spatially aware algorithms for clustering and DEA remain underutilized in ovarian biology, which likely reflects the field’s reliance on dissociation-based single-cell workflows.

In ovarian tissues, integrating spatial context offers significant advantages for clustering and subsequent cell-type or cell-state identification. In adult ovaries of both human and mice, cell identity in the ovary is intimately tied to location (Auersperg et al., 2001; Edson et al., 2009; Oktem & Oktay, 2008): granulosa layers surround the oocyte; theca cells form a distinct perifollicular boundary; stromal cells occupy specific cortical and medullary regions; and ovarian surface epithelium lies at the tissue periphery. Spatial information therefore provides powerful clues that complement transcriptional markers derived from traditional scRNA-seq studies, especially when gene expression alone may not fully distinguish closely related populations. Moreover, ST enables researchers to annotate follicle stages with unprecedented precision (Mantri et al., 2024; Martinez et al., 2025; Zhao et al., 2025a). Because follicles exhibit well-defined morphological transitions, histological context allows direct identification of primordial, primary, secondary, antral, and periovulatory follicles, something that dissociated single-cell methods cannot achieve. Nonetheless, spatial clustering is not without limitations. In practice, this may involve manipulating hundreds-of-gigabyte expression matrices and image stacks simultaneously, where tasks such as spatial neighbor graph construction can exceed the memory limits of standard desktop machines. This often demands High Performance Computing resources and memory-efficient workflows, which have been reported as bottlenecks (Martinez et al., 2025).

### Downstream analysis: trajectory inference

Trajectory inference has become a principal computational strategy for reconstructing developmental processes in the ovary, including folliculogenesis, reproductive cell development, and ovulation (Fan et al., 2019; Li et al., 2017). These algorithms order cells along a virtual continuum that reflects biological progression, enabling inference of transitions between cell states.

Trajectory inference can be broadly divided into two categories, pseudotime analysis and RNA velocity analysis. Pseudotime methods construct trajectories that reflects biological progression and infer transitions between cell states. Some of the most popular pseudotime tools include Monocle2/3 (Cao et al., 2019; Qiu et al., 2017; Trapnell et al., 2014), Slingshot (Street et al., 2018), and Palantir (Setty et al., 2019); these are the major choices across the iST and sST studies examined in this review (Saelens et al., 2019). Comparative benchmarking has evaluated their performance (Berge et al., 2020; Deconinck et al., 2021; Saelens et al., 2019), and Monocle2/3 remains the most widely adopted framework due to its outstanding performance, robustness, and interpretability. The choice of trajectory inference method, however, can vary depending on the type of trajectory expected. Additionally, many recent methods leverage spatial information (Heitz et al., 2025). Packages such as stLearn (Pham et al., 2023) and CASCAT (Yu et al., 2025) integrate physical proximity, tissue topology, or histological cues directly into trajectory construction.

In parallel with pseudotime reconstruction, RNA velocity infers the future transcriptional trajectory of individual cells by modeling the relative abundance of spliced and unspliced RNA. Tools such as Velocyto (Manno et al., 2018) and scVelo (Bergen et al., 2020) are therefore preferred when investigators aim to quantify dynamic transcriptional kinetics rather than static developmental ordering. Whereas trajectory inference reconstructs a developmental timeline from static snapshots, RNA velocity generates a short-timescale vector field that suggests where cells are moving transcriptionally, offering unique insight into rapid transitions. These analyses are valuable because they can distinguish cell states from transient intermediates, identify branching decisions, and pinpoint genes that drive rapid state transitions.

Despite these strengths, spatial trajectory inference remains underdeveloped in ovarian research, even though several spatially aware algorithms exist. To date, these methods have been applied primarily to tissues with relatively conserved spatial organization in adults, such as the brain, where the tissue architecture is highly conserved across individuals (Fjell & Walhovd, 2010). Their application to ovarian datasets is limited, primarily because the ovary poses a fundamentally different spatial problem, and fewer ovarian datasets have been generated. Unlike organs with stable architecture, the ovary undergoes dramatic, cyclic restructuring. Moreover, constructing a spatiotemporal trajectory typically requires sampling multiple animals or donors at different time points, and follicle spatial coordinates can vary widely across individuals. As a result, spatial location does not reliably correspond to developmental stage, and naïvely incorporating spatial coordinates into trajectory models may introduce noise rather than a biological signal. Consequently, spatially informed trajectory analyses in the ovary must be interpreted with caution and require more exploration by the field, including detailed validation studies. Researchers may need to process raw spatial coordinates, rely on within-follicle gradients rather than global tissue axes, or integrate multiple consecutive sections to mitigate the inherent variability of ovarian geometry.

### Downstream analysis: cell–cell interaction

Understanding ovarian physiology also requires deciphering the molecular dialogue among oocytes, granulosa cells, theca cells, stromal cells, endothelial cells, and immune populations. Cell–cell interaction (CCI) analysis that relies on ligand–receptor inference remains the principal computational framework for characterizing this communication landscape, specifically in scRNA-seq and spatial datasets (Dimitrov et al., 2022; Liu et al., 2022; Noël et al., 2021; Xie et al., 2023). CellChat (Jin et al., 2021) and CellPhoneDB (Efremova et al., 2020) are the most widely used in ovarian studies summarized in this review and these tools are very well developed, relying on validated and curated databases of ligand and receptor interactions. In addition to these dominant frameworks, NicheNet (Browaeys et al., 2020) has been used to link altered ligand–receptor activity to downstream transcriptional targets, which can be advantageous for hypothesis generation. Finally, CellTalkDB (Shao et al., 2021) has been used as a database for ligand–receptor interactions in several reproductive studies. When applied to scRNA-seq datasets, these tools provide a unique perspective on how CCIs may contribute to ovarian function and disease, and on which molecular signals are putative drivers. While powerful, these tools have historically not accounted for spatial location, which can limit interpretation because not all cell types can physically interact with one another in actual tissue.

To fill this gap, a new generation of spatially informed CCI inference tools has been designed to incorporate physical proximity directly into ligand–receptor modeling. Approaches such as COMMOT (Cang et al., 2023) and SpatialDM (Z. Li et al., 2023) incorporate spatial coordinates, adjacency, or spatial autocorrelation to refine interaction inference. This allows for interaction inference constrained by physical proximity, reducing spurious long-range predictions and improving the identification of niche-specific signaling events. Additionally, established frameworks are now evolving to incorporate spatial components. For example, CellChat2 now includes spatial weighting and distance-aware communication scoring, offering a hybrid model that combines its traditional ligand–receptor database with spatial constraints (Jin et al., 2025). As high-resolution imaging-based datasets become more common, spatially informed CCI analyses will likely play an increasingly central role in understanding the ovarian niche.

The need for spatially grounded CCI analysis is particularly acute in ovarian biology. Precise spatial relationships define the architecture of the ovary. Therefore, ST can provide critical contextual information, helping distinguish genuine follicle-specific interactions, such as oocyte–cumulus signaling. However, the spatial complexity demands exceptional caution. Since the ovary contains multiple follicles at different developmental stages, often in proximity to one another. Thus, their application must be guided by careful histological interpretation and follicle-level annotation.

### Downstream analysis: enrichment analysis and network inference

Downstream omics analyses often aim to elucidate the upstream regulatory logic and pathway-level mechanisms that drive ovarian physiology. To do this, several analytical approaches rely on either network-based analyses, which explore relationships among elements in a dataset, or enrichment analyses, which assess whether certain biological processes or predefined pathways are overrepresented in a dataset.

Transcription factor regulatory network inference and pathway-based enrichment analysis represent two complementary strategies for moving beyond descriptive clustering toward mechanistic interpretation. Transcription factor network inference aims to identify upstream regulators that drive cell state transitions, differentiation programs, or pathological remodeling by reconstructing transcriptional control architecture from gene expression data (Huynh-Thu et al., 2010). In parallel, pathway-based approaches, such as gene set enrichment and overrepresentation analysis, contextualize transcriptional signatures within known biological processes, enabling functional annotation (Subramanian et al., 2005). In their classical implementations, these methods operate solely on expression matrices and do not explicitly incorporate spatial coordinates or neighborhood structure. Tools such as SCENIC (Aibar et al., 2017; González-Blas et al., 2023; Sande et al., 2020) infer regulon activity from coexpression and motif enrichment, while enrichment frameworks, including clusterProfiler (Yu et al., 2012), Metascape (Zhou et al., 2019), GSEA (Subramanian et al., 2005), and fgsea (Korotkevich et al., 2021), evaluate pathway activation using databases such as Kyoto Encyclopedia of Genes and Genomes (Kanehisa & Goto, 2000), Gene Ontology (Ashburner et al., 2000), Reactome (Vastrik et al., 2007), and MSigDB (Liberzon et al., 2015), with ranked gene lists (Korotkevich et al., 2021; Subramanian et al., 2005). Because these approaches rely on gene-level statistics rather than spatial topology, they can be readily applied to spatial transcriptomics datasets without modification.

More recently, spatially informed packages have begun to emerge. Examples include SpaGRN (Li et al., 2025a), which integrates spatial adjacency with gene regulatory network reconstruction to identify spatially constrained relationships. In parallel, spatial autocorrelation statistics, such as Moran’s I (Moran, 1950), are increasingly applied to identify spatially variable genes, which can then serve as input for downstream enrichment or regulatory analyses that preserve spatial patterning. By incorporating proximity, spatial gradients, or neighborhood structure into regulatory inference, these approaches can distinguish intrinsic transcriptional programs from niche-driven regulation and uncover spatially restricted signaling domains that may be obscured in dissociated datasets. Despite these advances, ovarian studies to date have largely relied on expression-only implementations of regulatory and enrichment tools. This likely reflects the maturity, benchmarking, and interpretability of established single-cell frameworks, as well as the relatively recent adoption of high-resolution spatial platforms in reproductive biology. As spatial datasets become more prevalent, integration of spatially aware regulatory and pathway analysis may provide deeper mechanistic insight into how microenvironmental context shapes processes such as follicular development and ovarian remodeling.

## Spatial transcriptomics and its application in ovarian biology

Efforts to profile and understand the ovary using spatial methods have generated numerous resources and datasets that are crucial to the field, including those focusing on ovarian aging, follicle dynamics, and ovarian cancers. For this review, we summarize 40 studies performed on ovaries (Figure 3) that were collected from the literature as of writing in November to January 2026 (Bao et al., 2025; Bhattacharya et al., 2025; Chang et al., 2025; Denisenko et al., 2024; Dong et al., 2025; Ferri-Borgogno et al., 2023; Garcia-Alonso et al., 2022; Ge et al., 2025; Hsu et al., 2024; Huang et al., 2025; Jones et al., 2024; Kang et al., 2025; Kavarthapu et al., 2024; Kırboğa et al., 2025; Laury et al., 2024; Le et al., 2026; Li et al., 2024 ; Li et al., 2025b; Lin et al., 2023; Lu et al., 2024; Luo et al., 2024, 2025; Mantri et al., 2024; Martinez et al., 2025; Mu et al., 2025; Pan et al., 2025; Perez-Villatoro et al., 2026; Russ et al., 2022; Schweizer et al., 2025; Shi et al., 2023; Tai et al., 2025; Wamaitha et al., 2025; Winkler et al., 2024; C.-C. Wu et al., 2024; M. Wu et al., 2024; Yeh et al., 2024; Yu et al., 2024; Zhao et al., 2025a, 2025b). As over half focus on ovarian cancer, disease-driven research continues to lead methodological innovation and early adoption of spatial technologies. On the other hand, Visium remains the most widely used platform, accounting for over half of reported datasets, reflecting its accessibility, standardized workflows, and compatibility with existing single-cell analysis pipelines. Finally, the majority of studies pair spatial transcriptomics with scRNA-seq, highlighting the field’s recognition that spatial data are more powerful and convincing when anchored to single-cell references. Together, these resources and datasets have shed light on novel ovarian biology, identified cell states and interactions that drive cellular functions, and provided mechanistic insights into dynamic processes such as ovulation.

### Atlas resources for ovarian and gonadal biology

Overall, we surveyed five atlas studies and a central contribution is their ability to validate and refine canonical ovarian cell identities using spatially constrained expression patterns. In the human ovary, ST enabled precise, morphology-guided selection of adjacent follicular compartments and profiling of granulosa and theca cell layers (Jones et al., 2024). Using GeoMx spatial profiling, this approach revealed clusters that confirmed classical theca and granulosa cell markers in human samples (Hatzirodos et al., 2015; Skinner et al., 2008), including CYP17A1, CYP11A1, PTCH2, INSL3, APOE, BGN, and DHCR24, while also uncovering previously unrecognized theca-enriched genes such as S100A13, ALAS1, FDX1, and DLK1, for which FDX1 and ALAS1 sit directly within cholesterol acquisition and steroidogenic pathways (Jones et al., 2024).

High-resolution atlases spanning multiple embryonic and postnatal timepoints have resolved temporally and spatially restricted populations in mice, including premeiotic and meiotic germ cells marked by *Nanog*, *Dnd1*, and *Stra8*, oocytes marked by *Sycp3* and *Dazl*, pregranulosa and granulosa subtypes expressing *Foxl2*, *Kctd14*, *Fst*, *Inhba*, and *Ingbb*, and theca cells defined by *Cyp17a1*, *Cyp11a1*, and *Gli1* (Martinez et al., 2025). Beyond follicular compartments, this mice atlas systematically cataloged mesothelial cells (*Msln*, *Upk3b*), ovarian surface epithelium in humans (*UPK3B*, *KRT19*, *LHX9*), rete ovarii (*PAX8*, *GFRA1*, *RNASE12*), stromal fibroblasts (*Dcn*, *Lum*, *Col1a1*, *Col6a1*, *Pdgfra*), endothelial (*Pecam1*, *Cldn5*, *Emcn*), and lymphatic cells (*Prox1*, *Lyve1*), establishing a comprehensive spatial cell census of the developing mouse ovary (Martinez et al., 2025). Another developmental mice ovary study reported overlapping follicular and meiotic markers, such as *Inhbb* and *Fshr* in growing follicles and *Stra8* for entering meiosis (Zhao et al., 2025a). Importantly, this work extended prior model (Koubova et al., 2006) by identifying transcription factors, such as *Msx2* and *Zglp1*, as upstream regulators that promote meiosis initiation by activating *Stra8* (Zhao et al., 2025a).

Cross-species harmonized atlases (Marečková et al., 2022) integrating human and mouse gonadal development further demonstrated that, while overall spatial organization and lineage trajectories are conserved, key regulatory programs diverge between species. Notably, LGR5, a marker of second-wave pregranulosa cells in mice, is restricted to early-stage germ cells in humans, highlighting species-specific deployment of canonical regulators and underscoring the need for human–mouse aligned reference maps when interpreting ovarian development (Garcia-Alonso et al., 2022). In rat ovaries, integration of scRNA-seq and ST in mice resolved multiple granulosa and luteal states, including lipid metabolism-enriched luteal populations marked by *Fdx1*, *Fdxr*, and *Cybpa11*, which are difficult to distinguish transcriptionally without spatial context (Shi et al., 2023). Spatial localization further revealed that macrophages, B cells, and T/NK cells preferentially occupy the inner corpus luteum during regression rather than development, supporting a spatially restricted role for immune populations in corpus luteum decline and tissue remodeling (Shi et al., 2023). The role of immune cells in these processes is exciting and warrants further exploration. Together, these atlases demonstrate that spatial context is essential for linking cell identity, gene regulation, and tissue-level dynamics across various stages of ovarian development and the cycle. Despite progress, there is a need to create higher resolution maps that leverage true-single-cell technologies, leverage spatially aware trajectory inference methods, and better contextualize CCIs that drive ovarian development.

### Ovarian aging

Spatially resolved transcriptomic (ST) methods have played critical roles in understanding ovarian aging because of the tight link between aging phenomena and tissue architecture, where the latter cannot be analyzed postdissociation. In human ovaries, ST with Visium along with scRNA-seq delineated three granulosa cells and five theca-stromal cell types, characterized by their marker genes and spatial location (M. Wu et al., 2024). Among the spatially resolved canonical aging hallmarks, notably, FOXP1 emerged as a putative upstream regulator of ovarian cellular senescence in human. Activation of FOXP1 through quercetin delays ovarian aging, suggesting that its downregulation contributes to aging-associated decline (M. Wu et al., 2024).

In mice, ST was used to define the effects of aging within the follicles, especially along the radial dimension of granulosa cells (Russ et al., 2022). In antral follicles, ST with Visium enabled the definition of inner versus outer granulosa cells and revealed that aging selectively affects pathways for activin–follistatin signaling, transforming growth factor-β signaling, apoptosis, and gap junction communication in mice. These findings directly link granulosa–oocyte communication, as reflected in *Gja1* and *Gja4* gene expression, to aging and female reproductive failure, thereby resolving how aging perturbs these signaling pathways along defined follicular axes rather than uniformly across the ovary (Russ et al., 2022). In another mice study, spatial division of labor, where inner granulosa cells within follicles prioritize proliferation while outer cells specialize in hormone sensing and production, is progressively lost with age (Lan et al., 2026). Researchers profiled 21 mouse ovaries with 653 follicles and 234 corpora lutea with Slide-seq across the estrous cycle and chronological age (Lan et al., 2026). Aging was further found to disrupt the temporal coordination of folliculogenesis and corpus luteum clearance before the cessation of cycling, accompanied by spatially restricted immune dysregulation including chronic immune recruitment to persistent corpora lutea (Lan et al., 2026).

Moreover, ST has been critical for further connecting ovarian aging with mitochondrial dysfunction. Spatially informed analysis of granulosa/cumulus cells pinpointed ferredoxin FDX1, which is a marker of ovarian aging, as a regulator of energy metabolism in these particular cells in human (C.-C. Wu et al., 2024). Spatially informed interrogation of ferroptosis-related pathways, in turn, pinpointed region-specific disturbances in iron homeostasis, oxidative stress, and TP53-mediated iron death (Lin et al., 2023). These findings collectively validate that, unlike a homogeneous process, ovarian aging is region-specific, involving localized senescence, loss of communication, and metabolic vulnerability, and that ST is crucial to capture this complexity.

In cynomolgus monkeys, ST with Visium identified regions enriched for inflammatory, angiogenic, apoptotic, and senescence-associated signatures, which were disproportionately represented in aged ovaries and were termed “hotspots”(Lu et al., 2024). Aging occurs through these defined spatial “hotspots” rather than through global changes in gene expression, as an MT2-high area was discovered (Lu et al., 2024). However, given the limited spatial resolution of sST and the inherent heterogeneity between sections, caution is warranted in overinterpreting such domains as discrete aging niches without orthogonal validation, which would be the key next steps for many studies.

### Ovulation and follicle dynamics

Ovulation is a rapid, spatially coordinated process in which transcriptional programs evolve over hours and are tightly linked with follicular architecture, making spatial approaches uniquely well-suited for its study. Two complementary mouse studies captured an ovulation time course using different spatial resolutions. Using Curio Seeker, a spatiotemporal atlas of mouse ovulation across defined periovulatory stages while preserving follicular structure (Mantri et al., 2024). This approach revealed patterning within follicles during the preantral-to-antral transition, including coordinated expansion of inner *Inhbb*^+^*Kctd14*^+^ granulosa cells surrounding the oocyte and outer mural granulosa cells devoid of *Kctd14* expression. Spatial profiling further enabled investigation of stromal and mesenchymal dynamics, uncovering time- and space-sensitive events, such as mesenchymal cell proliferation bursts at 6 hr post-hCG (Mantri et al., 2024). It is the first study that leveraged whole-transcriptome spatial profiling; however, the spot-based resolution is limiting.

We have also published a high-resolution iST ovulation time course that integrated MERFISH with scRNA-seq to resolve transient cell states and cellular interactions at subcellular scales during ovulation (Huang et al., 2025). This approach yielded early- and late-transcriptional states across populations of granulosa, cumulus, stromal, theca, luteal, and immune cells while retaining native tissue context. This approach identified early and late transcriptional states across granulosa, cumulus, stromal, theca, luteal, and immune populations, with known markers such as *Sult1e1* (cumulus), *Inhbb* and *Amh* (granulosa), *Lhcgr* (luteal), *Dcn* (stroma), and *Cyp17a1* (theca) anchoring cell identities (Morris et al., 2022). Importantly, it also uncovered previously undescribed cluster-enriched genes, including *Zfp804a* in cumulus cells, *Oca2* in theca cells, *Pdzrn3* in stromal populations, and *Gm2a* in luteal cells, with validated spatial localization. Significantly, it uncovered an unintuitive heterogeneity of cumulus cells, including an early cumulus signature characterized by neuronal-related genes and a late signature characterized by genes involved in cholesterol metabolism or steroidogenesis.

Spatial context further illuminated the dynamics of stromal and theca cell heterogeneity post-LH surge. Distinct fetal theca-related clusters were identified, including a progenitor-like population expressing *Gatm*, *Nr2f2*, *Tcf21*, and a steroidogenic cluster enriched for *Lhcgr* (Wamaitha et al., 2025). These mesenchymal cells influence epidermal growth factor receptor signaling, angiogenesis, immune responses, and metal homeostasis, as well as spatially distributed myeloid cells that receive signals from stromal niches during remodeling. In addition, the study identified multiple granulosa populations occupying distinct cortical and medullary niches, and traced their differential contributions to primordial versus activated follicles (Wamaitha et al., 2025). This might explain why only medullary follicles acquire endocrine function, but not the follicles in the cortex.

### Ovarian cancer

Spatially resolved transcriptomics (ST) has been leveraged most extensively to study high-grade serous ovarian carcinoma (HGSOC), where genetic heterogeneity is closely linked to stromal and immune architecture (Bhattacharya et al., 2025; Denisenko et al., 2024; Ferri-Borgogno et al., 2023; Perez-Villatoro et al., 2026; Yeh et al., 2024). A study conducted on Visium slides showed that HGSOC is not merely polyclonal but also contains genetically and transcriptionally distinct areas, spatially separated from each other (Denisenko et al., 2024). Copy-number inference analysis demonstrated several copy-number-altered (CNA) subclones in each tumor, which are distinct in histological and cellular composition, including immune and stromal cell abundance (Denisenko et al., 2024). Further CosMx analysis has also demonstrated that, in human, particular subclones are selectively associated with C1QC+ and CXCL9+ macrophages, CCL5+ T cells, and IGHG1+ plasma cells, or alternatively with COL3A1+ fibroblasts, and that subclone-biased expression of ligands such as S100A8, SAA1, and CXCL10 is substantially involved in shaping these niches. Taken together, these findings underscore that ST reveals the tight coupling between subclonal architecture and the composition and signaling dynamics of the tumor microenvironment in HGSOC.

Stromal heterogeneity is another prominent dimension of spatial architecture in HGSOC. Tumors in short-term survivors were specifically enriched in spatially defined stromal areas expressing POSTN, CD36, and COL1A1, but were depleted of such areas in long-term survivors (Ferri-Borgogno et al., 2023). Protein-level validation confirmed elevated periostin and CD36 in spatially restricted stromal regions, implicating extracellular matrix remodeling, lipid uptake, and metabolic reprogramming as stromal programs associated with aggressive disease and therapy resistance (Ferri-Borgogno et al., 2023).

Immune organization in HGSOC is also spatially organized and significantly influenced by tumor cell–intrinsic programs. In immune-reactive areas, cancer cells expressing major histocompatibility complex class II genes colocalize with CD4^+^ and CD8^+^ T cells and antigen-presenting myeloid cells and show strong coupling to IFN-γ signaling, suggesting that tumor cells themselves can participate in antigen presentation and local immune activation (Perez-Villatoro et al., 2026). The immune evasion in HGSOC represents a composite, malignant cell-intrinsic transcriptional response driven by CNAs, including dysregulation of interferon signaling, chemokine pathways, and regulators such as BMP7 and RUNX1, thereby explaining why tumors with different levels of tumor-infiltrating lymphocytes do not uniformly respond to immunotherapy (Yeh et al., 2024).

Metastasis- and motility-associated genes such as FOSB, AMOTL2, and MT-CO2 were identified via Xenium analysis, alongside more stable signaling components, including PLEK, CFB, and ADGRB1, thereby linking spatial state transitions to functional tumor behavior (Kırboğa et al., 2025). Complementing this systems-level view, spatially resolved tumor and immune architectures are connected to clinical outcome, showing that nonrecurrence is associated with tumor expression of TFPI2 and PIGR and immune-region enrichment of NKG7, while recurrence is marked by increased Treg/CD8^+^ ratios and closer Treg proximity to cancer cells, highlighting how spatial gene expression and cell–cell organization jointly shape prognosis in HGSOC (Kang et al., 2025). Moreover, ST analysis identified a spatially localized tumor transcriptional module that was highly enriched for synaptic and neuronal signaling genes, as well as a subgroup of heavily platinum-treated patients with unexpectedly poor survival outcomes (Bhattacharya et al., 2025). This program colocalized with regions expressing neuronal markers such as TRPV1 and TUBB3, highlighting the growing importance of sensory fiber- and neuron-like signaling in modulating the HGSOC tumor microenvironment (Bhattacharya et al., 2025). In complementary studies, the utilization of artificial intelligence–powered mapping of the tumor environment enabled the definition of a prognostic tumor region highly enriched in JUN and stress-response pathway genes, in which tumor cells with elevated DNA repair gene expression colocalized with a lack of macrophage and plasma cells (Laury et al., 2024).

In addition to HGSOC, ovarian clear cell carcinoma (OCCC) exhibits a distinct spatial pattern. Spatial profiling showed that the oxidative phosphorylation and glycolysis pathways, with high epithelial–mesenchymal transition (EMT) scores, are confined to the center of OCCC tumors. Whereas, peripheral and invasive regions display increasing EMT accompanied by hypoxia, DNA damage, and inflammatory signaling such as tumor necrosis factor-α/nuclear factor-κB and interleukin-6/Janus kinase/STAT3 (Le et al., 2026). In early-stage carcinoma, spatial analysis further revealed progression from immune-mimicry to mesenchymal signals, with high expression of FN1 and SMA, which may be linked to clinical outcomes (Tai et al., 2025).

Beyond single-modality approaches, spatial multiomics has also provided insights into human ovarian cancer. Integrated spatial proteomic and transcriptomic analysis of serous borderline tumors and low-grade serous carcinoma identified invasion-associated drivers, including CLIC3, POSTN, ADAM15, SNCG, and NOVA2. Interestingly, NOVA2, which regulates FN1 splicing to promote tumor-stroma invasiveness, was detected only at the proteomics level, highlighting the value of multiomics (Schweizer et al., 2025). Finally, spatial profiling of serous tubal intraepithelial carcinoma identified proliferative, immunoreactive, mixed, and dormant precursor states, underscoring the importance of considering serous tubal intraepithelial carcinoma in addition to the current binary grading system (Chang et al., 2025).

Collectively, these studies illustrate why ST has been particularly transformative in ovarian cancer. By preserving tissue topology, ST enables simultaneous mapping of genetic heterogeneity, microenvironmental composition, and signaling interactions within the same specimen, which provides an integrative perspective that cannot be reconstructed from dissociated datasets alone. Moving forward, more efforts that utilize multiomic platforms to link spatial architecture with treatment responses may represent a major opportunity to translate ST into predictive biomarkers and therapeutically actionable targets.

## Conclusion

The ST technologies are poised to deeply expand and refine our understanding of ovarian biology. The ability to link spatial coordinates to transcriptomic data enables novel lines of inquiry, particularly when ST data are coupled with computational analysis approaches that provide tissue-level context. The wealth of ST data focused on ovarian biology has led to refined marker genes for ovarian cell types, revealed novel roles for cells within their tissue niches, and enabled more comprehensive analyses of ovarian cells across dynamic processes such as ovulation and development. Additionally, large-scale consortium efforts such as the Human Cell Atlas (Thul & Lindskog, 2018) and HuBMAP (Börner et al., 2025) have further accelerated progress by generating harmonized cell type annotations and reference atlases across tissues, including the ovary. These coordinated initiatives provide standardized frameworks that facilitate cross-study integration and improve reproducibility in spatial and single-cell analyses. Despite significant progress, our analyses suggest that there is a lack of healthy, human ovarian tissues that have been profiled to date, as most datasets in humans focus on cancer. The lack of healthy human references, particularly across age groups, poses challenges for better interpretation of disease states.

With the advent of ST datasets, however, broader challenges remain. No single ST platform is universally optimal for ovarian research, and the pros and cons of each platform must be considered when designing experiments (Table 1). The sST methods excel at transcriptome-wide mapping, but they are limited by mixed-cell capture and the need for deconvolution. This makes these platforms well-suited for discovery applications, trajectory mapping that leverages the full transcriptome, and initial large-scale c-tissue mapping studies. The iST platforms offer true single-cell and subcellular resolution, enabling detailed analysis of tissue architecture and rare cell states, yet require targeted panel design and robust image analysis. This makes iST platforms well suited for refining questions in ovarian biology, performing detailed analyses of specific cell types or tissue architectures, linking to matched scRNA-seq datasets to inform broader analyses, and probing key transcripts of interest. As ST matures, thoughtful alignment between biological questions, tissue scale, and platform choice will be essential to maximize interpretability and biological insight.

The hypotheses generated from these datasets require subsequent experimental validation and functional testing. Spatially resolved transcriptomics (ST) provides correlative evidence but cannot establish causality on its own. Validation approaches such as immunohistochemistry, smFISH, or RNAscope can confirm spatially resolved expression patterns at the protein or RNA level, while functional studies including genetic perturbation, in-vitro culture systems, and organoid models can test the biological significance of computationally inferred interactions and trajectories. These experimental follow-ups are critical for translating ST findings into mechanistic insight and clinical application.

An additional challenge is the analysis of ST datasets. A growing ecosystem of computational methods now enables joint analysis with scRNA-seq references, spatially aware clustering and trajectory mapping, spatially aware CCI, and many others. Frameworks such as Seurat (Butler et al., 2018), Tangram (Biancalani et al., 2021), and Cell2location (Kleshchevnikov et al., 2022) facilitate cross-modality mapping, annotation transfer, and probabilistic deconvolution, helping bridge differences in resolution and measurement chemistry. Future work should focus on improving the analyses of multimodal spatial assays that jointly profile RNA, protein, and chromatin to enable more complete and functionally grounded models of ovarian tissue organization. As ST matures within the context of reproductive biology, these integrative frameworks will become increasingly crucial for constructing harmonized, multilayered maps, and digital models that capture the full complexity of ovarian physiology.

## Data Availability

This review did not generate any datasets; all data discussed are available in the cited references.
